# Pseudo-Normalization of the T-wave During Stress and Its Relationship With Myocardial Ischemia: Evaluation by Myocardial Perfusion Single Photon Emission Computed Tomography (SPECT)

**DOI:** 10.7759/cureus.38428

**Published:** 2023-05-02

**Authors:** Juan Alan Fuentes Mendoza, Luis Mario Gonzalez Galvan, Carlos Alberto Guizar Sanchez, Juan Andres Pimentel-Esparza, Juan Fuentes Jaime, Jorge Antonio Cervantes-Nieto

**Affiliations:** 1 Cardiology, National Institute of Cardiology Ignacio Chavez, Mexico City, MEX; 2 Cardiology, Policlinica Integral del Bajio, Irapuato, MEX; 3 Nuclear Cardiology, National Institute of Cardiology Ignacio Chavez, Mexico City, MEX; 4 Internal Medicine, PEMEX Regional Hospital Salamanca, Salamanca, MEX; 5 Cardiology, PEMEX Regional Hospital Salamanca, Salamanca, MEX

**Keywords:** single photon emission computed tomography (spect), nuclear medicine imaging, myocardial injury, t-wave inversion, cardiac stress test, myocardial ischemia and infarction

## Abstract

Background

The T-wave alterations are suggestive of ischemia, among them there is the pseudo-normalization (positivization of the T-wave, previously negative, during stress exercise). Myocardial single photon emission computed tomography (SPECT) at rest and stress is usually performed with Technetium 99 (Tc-99), which has high sensitivity and specificity for the detection of ischemic heart disease.

In this study, we decided to investigate the patients who pseudo-normalized the T-wave in the stress test to correlate with the existence of ischemia diagnosed by myocardial perfusion study, specifically myocardial SPECT in perfusion and rest with Tc-99.

Methodology

T - wave pseudo-normalization patients who underwent a myocardial perfusion SPECT between January 2018 and June 2019 were included in this retrospective study. We analyzed 81 patients: 50 patients with pseudo-normalization of T-waves and 31 patients, as a control group, without pseudo-normalization. A descriptive analysis of the quantitative variables was performed using Student's t-test or Mann-Whitney U test, and for the qualitative variables, the χ2 test or Fisher's exact test was performed.

Results

The degree of ischemia according to the presence or absence of pseudo-normalization of the T-wave. The pseudo-normalization of the T-wave in the group without ischemia (48.4% vs. 36%), for the mild degree the proportions were the same (38.7% vs. 38%), the moderate degree it was slightly higher in the pseudo-normalization of the T-wave (9.7% vs. 18%) and severe (3.2% vs. 6%).

Conclusions

In this study, the relationship between pseudo-normalization of the T waveform and ischemia, predominantly moderate to severe, was demonstrated. However, it was not statistically significant due to the size of the sample studied.

## Introduction

The T-wave on the electrocardiogram (T-ECG) represents the repolarization of the ventricular myocardium. Its morphology and duration are commonly checked to diagnose pathologies and assess the risk of life-threatening ventricular arrhythmias. However, the physiological background of the T-wave is not fully understood, making reliable interpretation of the T-wave difficult [1-3]. T-wave is usually positive, except in aVR and often in V1. Occasionally, T-wave inversion is also seen in anterior and inferior leads. A transmural repolarization gradient can explain this concordance with earlier repolarization occurring in the epicardium [4-6]. Among the alterations of the T-wave that are suggestive of ischemia, there is pseudo-normalization, or in other words, the positivization of a previously negative T-wave during a stress test [7,8]. However, some studies report that, in a previously infarcted heart, this same pseudo-normalization can be associated with viable myocardium or recovery of segmental mobility [9-11]. Nevertheless, results can be disparate when the sensitivity and specificity to predict myocardial recovery are low [12]. One important point to be kept in mind is that the studies performed for detecting ischemia in patients who normalized the T-wave after exercise was carried out nearly 30 years ago, with several diagnostic limitations. Myocardial single photon emission computed tomography (SPECT) at rest and stress is usually performed using Technetium 99 (Tc-99), which has high sensitivity and specificity for the detection of ischemic heart disease [13-18]. Considering these points, we decided to study the patients who pseudo-normalized the T-wave during the stress test, to correlate with the existence of ischemia diagnosed by myocardial perfusion study, specifically using myocardial SPECT during stress and rest, using Tc-99.

## Materials and methods

We conducted a retrospective observational study of all the patients who had T-wave pseudo-normalization and had undergone a myocardial perfusion SPECT study at the National Institute of Cardiology Ignacio Chavez, between January 2018 and June 2019. We included patients of both genders, over 18 years of age, who had a previous negative T-ECG, and who had undergone a myocardial SPECT stress test. We excluded patients who had a recent history of myocardial infarction (12 months), a history of ventricular arrhythmias, pregnant women, and patients under 18 years of age.

A descriptive analysis of the quantitative variables was performed. Depending on their normality, which was corroborated by the Shapiro-Wilk test, they were reported using mean and standard deviation, if the values were parametric, or using median and interquartile ranges if the values were non-parametric. In the same way, considering the normality, a bivariate analysis was done for quantitative variables using Student’s t-test or Mann-Whitney U test, as appropriate.

Qualitative variables were described using frequencies and percentages, and χ2 test or Fisher’s exact test was used for their bivariate analysis, depending on the number of events collected. Logistic regression models were built to identify the risk factors associated with ischemia (dependent variable) during the myocardial perfusion study. In all analyses, a value of <0.05 was considered significant.

## Results

In this study, 81 patients were included, of whom 69.1% were men, and 30.9% were women. The median age of the participants was 62 years. The most frequent comorbidities found in the participants were: previous myocardial infarction (61.7%), systemic arterial hypertension (53.1%), diabetes mellitus (38.3%), and smoking (37%). The rest of the baseline demographic variables are shown in Table 1.

**Table 1:** Demographic characteristics of patients with SPECT studies during 2019. IR= Interquartile range, SPECT - single photon emission computed tomography

Variable	Total (n = 81)	
	n	%
Women	25	30.9
Men	56	69.1
Diabetes mellitus	31	38.3
Systemic arterial hypertension	43	53.1
Dyslipidemia	17	21
Previous myocardial infarction	50	61.7
Obesity	16	19.8
Angina	30	37
Smoking	30	37
Variable	n	Median (IR)
Age (years)	81	62 (54 – 67)
Creatinine (mg/dL)	27	0.9 (0.76 – 1.0)
Potassium (mEq/L)	16	4.2 (3.75 – 4.4)

A subgroup analysis of the presence or absence of pseudo-normalization of the T-wave revealed that distribution by sex had no significant differences (p=0.32). On the other hand, the presence of diabetes mellitus was uniformly and equally distributed without any substantial differences, as were dyslipidemia, previous myocardial infarction, obesity, and smoking. It should be noted that differences were found between both groups in the distribution of arterial hypertension and angina. The rest of the information about the analysis is presented in Tables 2, 3.

**Table 2:** Demographic characteristics of patients who underwent SPECT studies, classified as per the presence or absence of pseudo-normalization of the T-wave. SPECT - single photon emission computed tomography

Variable	Absence of pseudo-normalization of the T-wave (n=31)		Presence of pseudo-normalization of the T-wave (n=50)		P
	n	%	n	%	
Women	11	35.5	14	28	0.32
Men	20	64.5	36	72	
Diabetes mellitus	15	48.4	16	32	0.14
Systemic arterial hypertension	21	67.7	22	44	0.03
Dyslipidemia	8	25.8	9	18	0.40
Previous myocardial infarction	17	54.8	33	66	0.31
Obesity	5	16.1	11	22	0.51
Angina	6	19.4	24	48	0.00
Smoking	11	34.5	19	38	0.82

**Table 3:** Demographic characteristics (age) of patients who underwent SPECT studies. IR= Interquartile range, SPECT - single photon emission computed tomography

Variable	Absence of pseudo-normalization of the T-wave (n=31)		Presence of pseudo-normalization of the T-wave (n=50)		P
	n	Median (IR)	n	Median (IR)	
Age (years)	31	61.5 (54-68)	50	63 (53-67)	0.84

**Figure 1:**
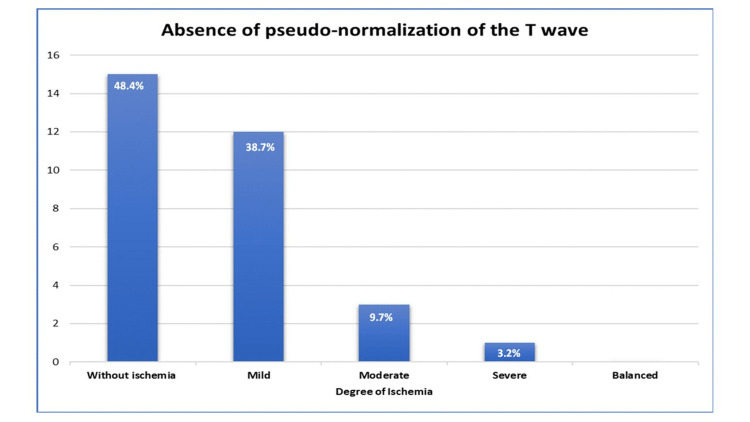
Degree of ischemia in absence of pseudo-normalization of T-wave in SPECT studies. SPECT - single photon emission computed tomography

**Figure 2:**
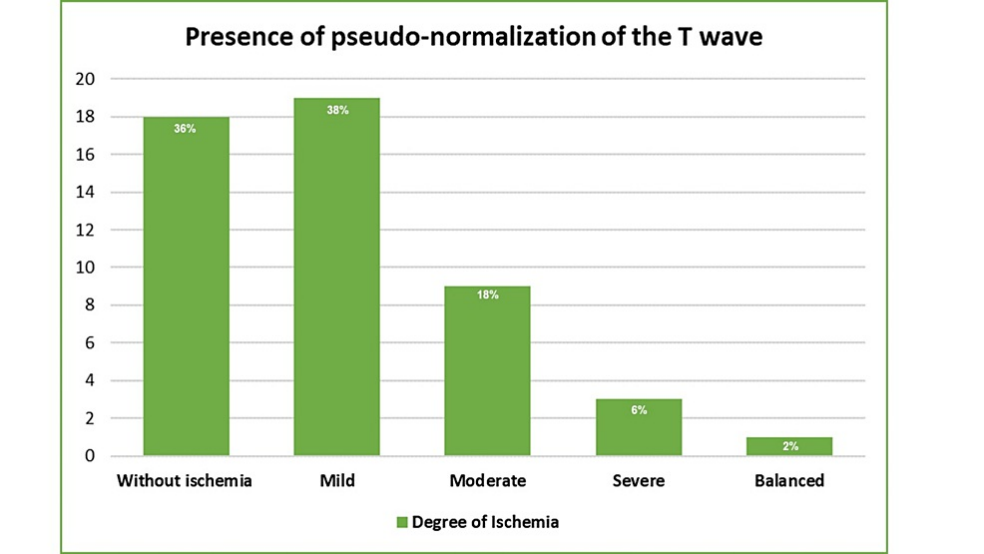
Degree of ischemia in presence of pseudo-normalization of T-wave in SPECT studies. SPECT - single photon emission computed tomography

Continuing with the analysis by subgroups, the degree of ischemia was analyzed based on the presence or absence of pseudo-normalization of the T-wave. It was found that there was a higher proportion of lack of pseudo-normalization of the T-wave in the group without ischemia (48.4% vs. 36%), while in the group with the milder degree, the proportions were the same (38.7% vs. 38%). In the pseudo-normalization group, it was slightly higher for the moderate (9.7% vs. 18%) and severe (3.2% vs. 6%) degrees. Despite this, in the final analysis, no statistically significant differences were found (p=0.71), as seen in Figures 1, 2.

A comparison between the presence of infarction revealed by the SPECT study and the pseudo-normalization of the T-wave showed that there was a more significant presence of infarction, proportionally speaking, in patients with pseudo-normalization of the T-wave (64% vs. 51.6%). In addition, in this same group, there was a lower prevalence of absence of infarction (36% vs. 48%). However, in the final analysis, no statistical significance was found (p= 0.27), as shown in Table 4.

**Table 4:** Finding of myocardial infarction by SPECT study in patients with pseudo-normalization of the T-wave. SPECT - single photon emission computed tomography

Variable	Total		Absence of pseudo-normalization of the T-wave (n = 31)		Presence of pseudo-normalization of the T-wave (n = 50)		P
	n	%	n	%	n	%	
Absence of infarction	33	40.7	15	48.4	18	36	0.27
Presence of infarction	48	59.3	16	51.6	32	64	

A descriptive analysis was performed in the population according to the myocardial perfusion study’s report of presence or absence of ischemia. In this analysis, it was found that there were no differences in terms of sex, arterial hypertension, dyslipidemia, obesity, and angina. The same was the case with smoking, age, creatinine, and potassium. However, the diabetic population did have differences in the presence of ischemia (52.1% in diabetics vs. 18.2% in non-diabetics). The same was the case with the population who had previous infarction (77.1% in previously infarcted vs. 39.4% in previously non-infarcted). The rest of the analysis results are given in Tables 5, 6.

**Table 5:** Demographic characteristics of patients in SPECT study according to the presence or absence of ischemia. SPECT - single photon emission computed tomography

Variable	Without ischemia (n = 33)		With ischemia (n = 48)		P
	n	%	n	%	
Women	12	36.4	13	27.1	0.37
Men	21	63.6	35	72.9	
Diabetes mellitus	6	18.2	25	52.1	0.01
Systemic arterial hypertension	18	54.6	25	52.1	0.82
Dyslipidemia	5	15.2	12	25	0.28
Previous myocardial infarction	13	39.4	37	77.1	0.01
Obesity	5	15.2	11	22.9	0.38
Angina	12	36.4	18	37.5	0.91
Smoking	14	42.4	16	33.3	0.40
Pseudo-normalization of the T-wave	18	54.6	32	66.7	0.27

**Table 6:** Demographic characteristics of patients in SPECT study according to the presence or absence of ischemia (Continuation of Table 5). IR= Interquartile range, SPECT - single photon emission computed tomography

Variable	Without ischemia (n = 33)		With ischemia (n = 48)		P
	n	Median (IR)	n	Median (IR)	
Age (years)	48	63(56-67.5)	33	60(51-67)	0.27
Creatinine (mg/dl)	9	1(0.9-1)	18	0.9(0.7-1)	0.49
Potassium (mEq/L)	4	4(3.6-4.6)	12	4.2(3.9-4.4)	0.8

A logistic regression model was performed to predict ischemia during the myocardial perfusion study. This analysis revealed that the factors that had the most power to predict ischemia were a history of diabetes mellitus (OR 4.66, 95%; CI 1.57-13.84) and a history of previous myocardial infarction (OR 5.81, 95%; CI 1.92-17.57). Although the other variables exhibited a trend of having the power to predict ischemia, they did not have statistical significance: dyslipidemia (OR 1.68, 95%; CI 0.52-5.44), obesity (OR 1.6 95%; CI 0.49-5.2), and pseudo-normalization of the T-wave (OR 1.6 95%; CI 0.63-4.06), as shown in Table 7.

**Table 7:** Logistic regression model adjusted for age and sex for the prediction of ischemia during the SPECT study. SPECT - single photon emission computed tomography

Variable	OR	CI 95%	p
Diabetes mellitus	4.66	1.57-13.84	0.00
Systemic arterial hypertension	0.82	0.32-2.08	0.68
Dyslipidemia	1.68	0.52-5.44	0.38
Previous myocardial infarction	5.81	1.92-17.57	0.00
Obesity	1.60	0.49-5.20	0.43
Angina during test	1.18	0.45-3.06	0.72
Smoking	0.67	0.26-1.72	0.41
Pseudo-normalization of the T wave	1.60	0.63-4.06	0.31

**Figure 3:**
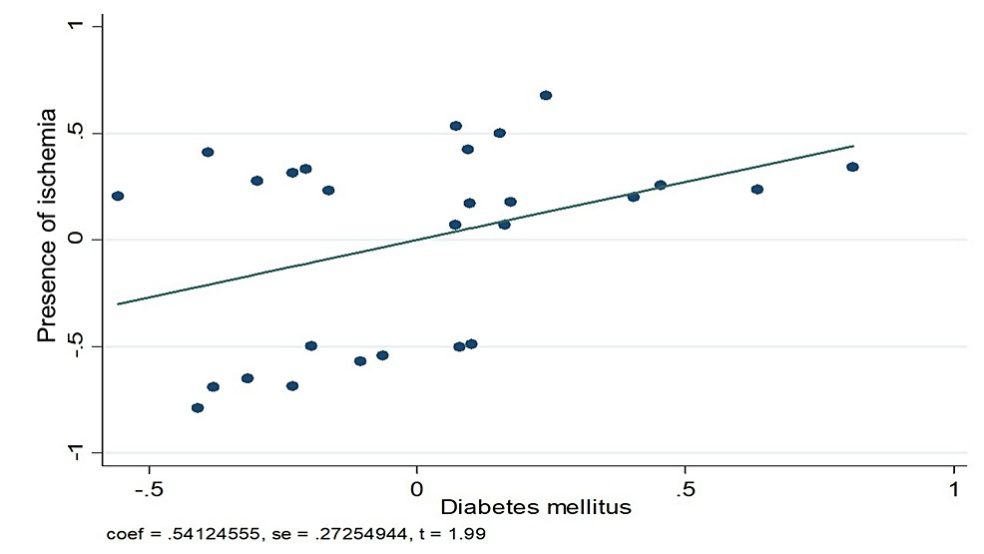
Linear relationship between the presence of ischemia by SPECT study and type 2 diabetes mellitus. SPECT - single photon emission computed tomography

**Figure 4:**
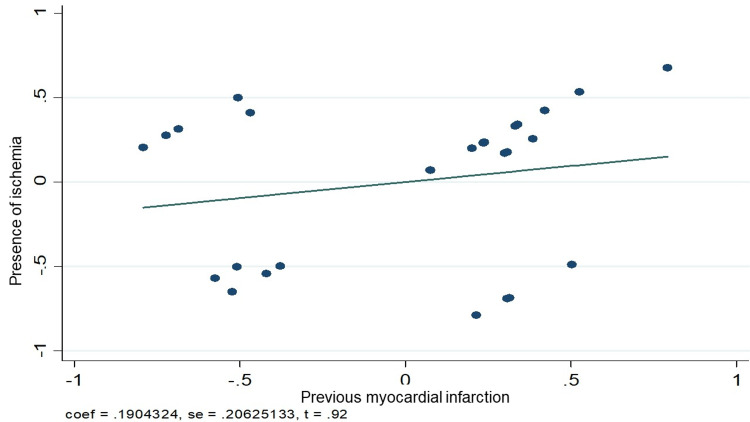
Linear relationship between the presence of ischemia and previous infarction by SPECT study. SPECT - single photon emission computed tomography

**Figure 5:**
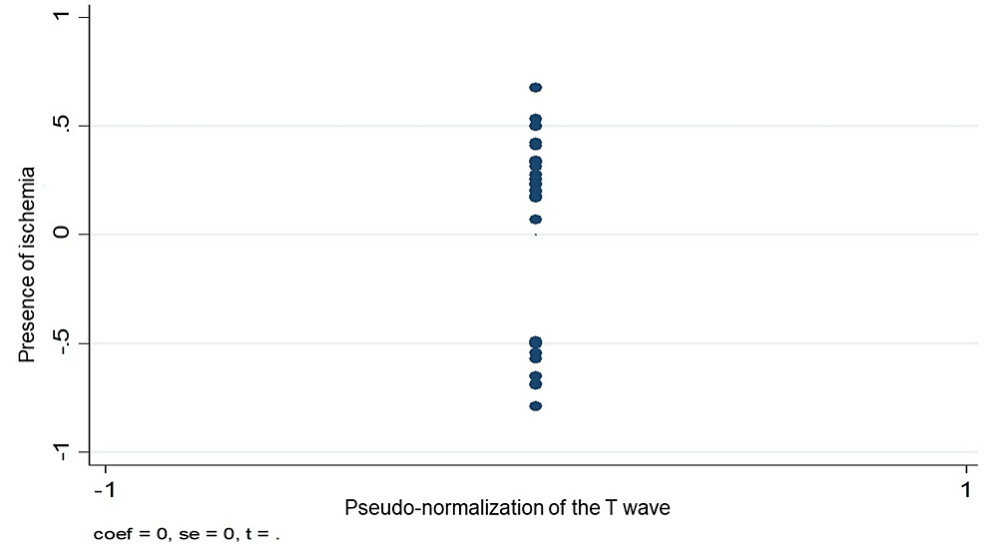
Linear relationship between the presence of ischemia and the appearance of T-wave pseudo-normalization by SPECT study. SPECT - single photon emission computed tomography

Figures 3-5 graphically demonstrate the relationship between some of the variables and the presence of ischemia. Figure 3 clearly shows how the presence of diabetes mellitus has a positive relationship with the presence of ischemia. On the other hand, Figure 4 shows the same positive relationship between a history of infarction and ischemia in the myocardial perfusion study. Finally, in Figure 3, it can be seen how pseudo-normalization was not related to the presence of ischemia.

## Discussion

In this cross-sectional study, we sought to determine if there was a relationship between the pseudo-normalization of the T-wave and the presence of myocardial ischemia, as previously described by Lavie et al. in 1988 [8]. In the subgroup analysis (pseudo-normalization vs. no pseudo-normalization), it was found that the variables were distributed homogeneously; however, there were specific differences in patients who had arterial hypertension and angina pectoris.

The presence of ischemia (moderate and severe) in this study was numerically more frequent in the group of patients with pseudo-normalization of the T-wave during stress. However, the p-value could have been more statistically significant if we had recruited more patients. Notably, there may be a correlation between ischemia and pseudo-normalization of the T-wave, which was more frequent in patients with moderate to severe ischemia, suggesting that when pseudo-normalization occurs and ischemia is present, it is more likely to be moderate to severe. Similarly, myocardial infarction was more frequent in those with pseudo-normalization but without statistical significance.

A logistic regression model revealed that pseudo-normalization of the T-wave increased the probability of the presence of ischemia by 1.6 times. The vast confidence intervals indicate the need for a larger sample to attain statistical significance. However, we should keep in mind, the increase in the OR between pseudo-normalization and the presence of ischemia.

## Conclusions

This study demonstrated the relationship between pseudo-normalization of the T-wave and ischemia, predominantly moderate to severe, was demonstrated. However, the limitation of our study is the small sample of patients that we were able to include, which is why we believe it was not statistically significant.
